# Empowering People with Parkinson’s: Reframing Self-Management in Parkinson’s—A Critical Reflection of Current Practice

**DOI:** 10.3390/healthcare13212673

**Published:** 2025-10-23

**Authors:** Julie Jones, Bhanu Ramaswamy

**Affiliations:** 1 School of Health, Robert Gordon University, Aberdeen AB10 7AQ, UK; 2 School of Health and Social Care, Sheffield Hallam University, Sheffield S10 2BP, UK; b.ramaswamy@shu.ac.uk

**Keywords:** Parkinson’s, self-management, enablement, empowerment, person-centered approach

## Abstract

**Background:** Parkinson’s is among the fastest-growing neurological disorders, characterised by motor and non-motor symptoms that affect daily function and quality of life. With no cure, sustainable management strategies are essential. Self-management is a key component, enabling people with Parkinson’s to actively manage symptoms, treatment, and lifestyle, reflecting the wider long-term conditions (LTCs) approach to improving outcomes and well-being of people affected by these conditions. However, more than half of people living with Parkinson’s (PwP) report difficulties in engaging with self-management, often due to limited knowledge, confidence, or access to tailored interventions. **Aims:** This paper explores the theoretical underpinnings, key drivers, and current evidence base for self-management in Parkinson’s. It examines the relevance and limitations of applying LTC models to a progressive and highly individualized condition such as Parkinson’s. Despite global guideline recommendations, self-management support remains a significant unmet need. While self-management has the potential to improve adherence, symptom control, and activity levels, uncertainties remain about what constitutes effective, meaningful support. There is a need for a nuanced, person-centered approach embedded within integrated care systems. **Conclusions:** To date, self-management has not demonstrated sustained benefits for PwP, in part due to limitations in how current models are conceptualized and delivered. This paper highlights the challenges of existing approaches and proposes a new framework that enables and empowers PwP and their support networks to live well with Parkinson’s. Rooted in partnership, enablement, and co-production, the proposed model promotes the development of personalized toolkits of strategies that help individuals navigate and mitigate the challenges of life with Parkinson’s. This reframing has important implications for future research, clinical practice, and policy.

## 1. Introduction

Parkinson’s is the fastest-growing, incurable neurodegenerative disorder worldwide [1]. The complex and evolving interplay of motor and non-motor symptoms significantly impact daily functioning and quality of life requiring a comprehensive, sustainable approach to condition management.

Self-management, as an intervention, has emerged as a critical component in other long-term conditions (LTCs), providing individuals agency in managing their symptoms, treatment, and lifestyle choices. Supporting and empowering people to take responsibility for their own condition improves health outcomes, psychological well-being, and overall quality of life [2]. For people living with Parkinson’s (PwP) as well as their families, carers, and support networks, self-management can enhance medication adherence, facilitate symptom control, promote engagement in physical activity, and ultimately contribute to slower functional decline and to improved quality of life, although substantial uncertainty remains regarding the optimal content, delivery, and timing of self-management interventions [3]. Conversely, a lack of appropriately timed and sufficient information [4], has been reported to lead to demoralization in those not provided a message of hope and a way to self-manage aspects of the condition [5]. The World Health Organization (WHO) plus individual country health strategies recommend access to education and self-management support as part of any rehabilitation intervention, yet it remains a significantly unmet need within the Parkinson’s community [6,7].

The importance of multi-disciplinary intervention and the availability of rehabilitation programs with interventions for individuals with Parkinson’s as a core feature from diagnosis onward cannot be emphasized sufficiently; however, given the scope of this article, the focus remains on aspects of self-management education.

While studies have explored self-management approaches adapted from other long-term conditions, many have yielded limited positive outcomes and have not comprehensively evaluated their impact on health, behavioural, or related outcomes. This highlights the need for further research to develop and evaluate self-management strategies that effectively support people with Parkinson’s disease in living well with their condition.

The aim of this opinion piece is therefore to explore the concept of self-management in Parkinson’s, outlining its theoretical underpinnings, key facilitators, and current evidence base. It also addresses existing challenges, emphasising the importance of personalized, integrated models that move beyond individual responsibility to embed self-management within supportive systems of care. In doing so, we propose a dynamic, person-centered framework for self- and shared management with implications for future research, policy development, and clinical practice.

## 2. What Is Self-Management?

The growth in numbers of people living with a LTC has resulted in a rise of global health strategies and policies advocating mechanisms which enhance independence, supporting people in maintaining their own health, so reducing healthcare utilization whilst maintaining a quality of life (QoL) [7,8].

Self-management is a cornerstone of LTC care, positioning individuals as active participants in maintaining their health, function, and overall well-being. This concept is deeply rooted in the Chronic Care Model [9], which emphasizes productive interactions between informed, activated individuals and proactive healthcare teams to achieve improved health outcomes. The model was investigated for the impact of acquisition and application of the knowledge, and skills in building confidence to manage the medical, emotional, and lifestyle-related aspects of living with LTCs [10].

Self-management principles are grounded in empowerment and shared responsibility, fostering a collaborative partnership between individuals and healthcare professionals [11]. Lorig and Holman’s skills-based framework proposes five core self-management processes: problem-solving, decision-making, resource utilization, the formation of patient-provider partnerships, and the ability to take action [10]. These elements are essential in enabling individuals to navigate the complexities of chronic illness while maintaining autonomy and psychological well-being.

For the purposes of this opinion piece, we adopt the definition of self-management proposed by Lorig (2003, p. 11 [10]): “*the knowledge and skills required to maintain an active and emotionally satisfying life in the face of a chronic condition.*” This definition provides a guiding framework for evaluating the literature and identifying key components of effective self-management in Parkinson’s.

## 3. Drivers for Self-Management in Parkinson’s

Several converging factors have contributed to the growing emphasis on self-management in Parkinson’s, reflecting wider changes in healthcare delivery and patient engagement. The shift towards patient- or person-centered care recognizes the expertise of individuals living with LTC’s and seeks to involve them as active partners in managing their health. In Parkinson’s, where symptoms and progression vary significantly between individuals, a person-centered approach allows care to be tailored to personal goals, values, and preferences [4,12], making self-management both a necessity and an opportunity for empowerment.

Global healthcare approaches play a critical role in promoting self-management as a strategic response to the rising prevalence of Parkinson’s [6] positioning self-management as key to managing health outcomes. Programmes of intervention advocate for accessible education, behaviour change support, and ongoing professional collaboration to enable individuals to manage their condition more effectively. Complementing these efforts, digital health tools-including symptom trackers, medication reminders, telehealth platforms, and virtual exercise programmes-offer innovative avenues for delivering personalized support, particularly in under-resourced or remote settings.

Patient advocacy and support organisations (including each country’s main Parkinson’s charity), plus similar international bodies such as the Michael J Fox Foundation and European Parkinson’s Disease Association, have been instrumental in advancing the self-management agenda. The peer support systems and awareness-raising of the lived experience of Parkinson’s contribute to research and policy development, to resource development, and campaigning.

These drivers towards self-management as a shared journey rather than an individual burden highlight the importance of embedding self-management within a supportive ecosystem—one that combines person-centered care, enabling policies, technological innovation, and community solidarity to help people with Parkinson’s live well.

## 4. Current Evidence Base for Self-Management in Parkinson’s

Two systematic reviews [13,14] have explored self-management interventions in Parkinson’s; Pigott et al. focuses on effectiveness [13], whilst Tuijt et al. provides complementary insights into the perceptions and experiences of self-management, offering a richer understanding of potential practice interventions [14].

Pigott et al.’s review of 36 studies—primarily conducted in North America and Europe—included over 2800 participants and examined the impact of self-management interventions on quality of life, well-being, and functional outcomes [13]. Over half of the studies were randomized controlled trials (RCTs), and a third employed pre- and post-test designs. Findings were constrained by methodological heterogeneity, high risk of methodological bias and limitations in reporting standards.

The Stanford Chronic Disease Self-Management Programme (CDSMP), Patient Education Program Parkinson, and adaptations such as “Strive to Thrive” were the most frequent self-management programmes studied. Interventions varied widely in content, delivery, and professional involvement, limiting synthesis and resulting in a meta-analysis of just four studies. Although the effect on QoL was small and non-significant (Hedges’ g = −0.17, *p* = 0.38; I^2^ = 68%), the review highlighted that integration of education and skills-based strategies were the most promising components of self-management interventions. Multicomponent self-management programmes integrating physical activity, cognitive behavioural therapy, mindfulness, and symptom self-monitoring were associated with better outcomes than unidimensional education-based interventions. These findings echo prior work [3,10,15], emphasising the need for self-management programmes that encompassed problem-solving, decision-making, resource utilization, forming partnerships with healthcare providers, and taking action.

Tuijt et al.’s qualitative synthesis of six high-quality studies identified seven self-management components valued by PwP: medical management, physical activity, self-monitoring, psychological strategies, independence, social engagement, and tailored education [14]. Crucially, PwP preferred programmes that provided not just knowledge, but actionable strategies such as practical functional skills, problem-solving capabilities, and contextualized education, offering more of a tool kit approach to delivery rather than reliance on information provision, providing PwP with both the “what” and the “how”.

This second review highlighted the pivotal role of Parkinson’s-informed healthcare professionals (HCPs) in effective intervention delivery. Staff who were knowledgeable, motivational, and supportive—enabling personalization and shared decision-making—were particularly valued by PwP as were programmes adopting a partnership approach to delivery [3,16]. Social support and group-based delivery were highlighted as important, with group interventions linked to more positive outcomes. Peer support facilitated the normalization of experiences, offered emotional reassurance, and enabled social learning—mechanisms that reinforce behaviour change strategies such as action planning and self-monitoring [17].

The “Strive to Thrive” programme [18], an adaptation of the CDSMP encompassing a Parkinson’s-specific week, showed benefits in physical activity, relaxation, and spousal support. However, self-efficacy in PwP declined post-intervention, potentially reflecting the limited Parkinson’s-specific content or increased self-awareness of the challenges of living with Parkinson’s. Similarly, Park et al. trialed a digital self-management intervention using wearable technology and app-based feedback [19]. While non-motor symptoms and self-efficacy improved, perceived self-management ability did not-possibly due to insufficient skills training in the remote format.

The mixed findings across varied studies highlight the complex and multidimensional nature of self-management with most research prioritizing effectiveness over understanding lived experience. Despite clear drivers for self-management, current research has been unable to determine either the optimal content or effectiveness over time.

## 5. Challenges of Self-Management in Parkinson’s

Self-management in Parkinson’s presents multiple challenges, stemming from the highly individual and evolving nature of the condition. PwP experience a wide spectrum of symptoms and disease progression, meaning their self-management needs vary significantly—not only between individuals but also for the same person over time. However, many current self-management programmes offer a static, one-off educational intervention that lacks responsiveness to these changing needs. Furthermore, such programmes often prioritize the provision of information over the development of practical strategies to promote independence and autonomy in daily life.

The lack of an agreed vocabulary to distinguish the different ways in which self-management can be applied has led to poor conceptual clarity around the term “self-management” itself. This creates inconsistency in what educational interventions should include, when they should be delivered throughout the disease trajectory, how frequently, and whose perspectives (clinicians, patients, carers) they are shaped by [3,13,16,20,21]. As a result, programme content, delivery methods, and outcome measures vary widely. Differences include group versus individual delivery, whether carers are involved as well, mode of delivery (in-person or digital), and cost-effectiveness considerations [21,22].

Another key challenge lies in the implementation and understanding of deliverer competencies. Skills such as motivational coaching—essential for supporting behavioural adaptation—are inconsistently applied and poorly described in the literature [23]. Moreover, most studies evaluate self-management interventions using short-term pre–post measures, typically conducted within a few years of diagnosis. Longitudinal follow-up is rarely included, meaning we lack evidence on whether the education provided remains relevant as the condition progresses, or whether further support is needed to address new or evolving challenges.

Where programmes are delivered through statutory healthcare services, there is an inherent tension between addressing what clinicians can provide within service constraints and what individuals with Parkinson’s may need [16]. In contrast, third-sector initiatives (as provided through non-Governmental or non-profit organizations) such as Parkinson’s UK’s “First Steps”—co-designed and delivered by people affected by the condition—demonstrate the potential of peer-led models to bridge this gap [17].

Additionally, studies focusing on PwP from ethnically diverse backgrounds highlight the importance of including culturally relevant content. Religion, community networks, family support, and the formation of new peer connections have all been identified as important factors influencing engagement with self-management [24,25,26]. As few people from marginalized communities sign up to traditionally provided self-management programs, we lack an understanding as to what issues specific to these communities would be considered essential additions to these programs as different to aspects currently provided. These findings highlight the need for more person-centered, culturally sensitive, and adaptable approaches that recognize the heterogeneity of experiences and preferences among those living with Parkinson’s.

## 6. What People with Parkinson’s Want from Self-Management

Over the past decade, both authors’ work with local groups has highlighted the sense of disempowerment reported by people from the point of diagnosis, which contributes to difficulties in coping with the variability of symptoms [27,28]. This highlights how the point of diagnosis is a crucial moment when key services need to be introduced to facilitated and enable PwP to self-manage and live well with their Parkinson’s.

The lack of timely intervention however is not uncommon with over half of PwP reporting difficulties in self-management, often due to limited knowledge, confidence, or access to tailored resources [4,29] or in implementing effective self-management strategies due to insufficient support, limited health literacy, or a lack of personalized interventions [30].

Emerging research indicates that PwP and their family and friends value opportunities that support a sense of control over the condition. In 2016, Ramaswamy worked with a group of stakeholders affected by Parkinson’s who requested a reconceptualization of the way in which health care professionals (HCPs) dealt with the condition [28]. They expressed an importance in the recognition of how their personal narrative could lead to a more collaborative approach to management—whether through partnerships with HCPs, peer networks, or members of their immediate support system.

**Figure 1:**
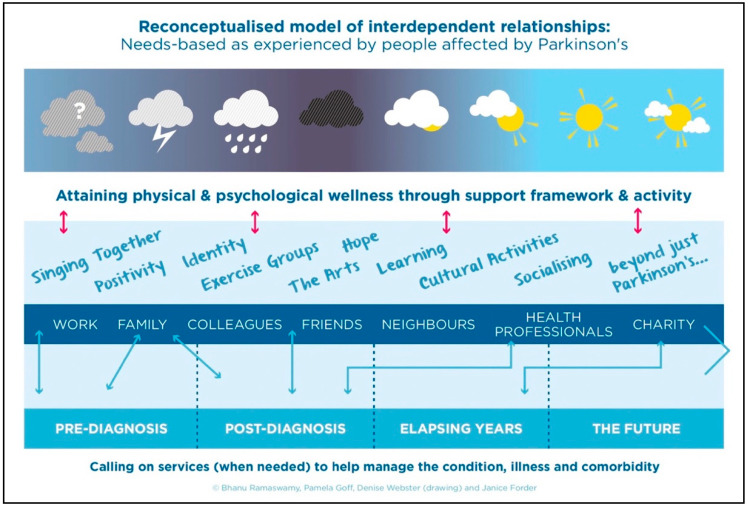
Ramaswamy illustrates a Social Model created by people affected by Parkinson’s [28] (used with authors permission).

Figure 1 was the stakeholder group’s attempt to show that whilst the course of the condition might follow a linear route of diagnostic subsections described by HCPs (at the bottom of the diagram), they learned that relationships from engaging with wider support networks helping them share the management, and burden of the diagnosis—it literally allowed them to negotiate through an initial cloud-filled world full of questions, through the darkest moments of diagnosis and out to a far sunnier and manageable world. In some contexts, such as within certain ethnic communities or when cognitive limitations are present, participants expressed the importance of having a trusted proxy to advocate on their behalf and help articulate their needs [4,5].

PwP emphasized how the progressive nature of Parkinson’s and fluctuating symptoms (in severity and ways in which these are experienced) over time required a programme or education co-designed by people affected by Parkinson’s delivered at different stages of the condition and more personalized to their different phenotypes, coping styles and their support networks [3,31,32].

Current research highlights that the role of HCPs needs to change to accommodate such needs [4,5]. At diagnosis, a period of uncertainty and anxiety, regular health staff input was valued, with this dependency declining with time as PwP developed a sense of self-efficacy, enabling them to develop a sense of control [27,28] (Jones and Williams 2023, Ramaswamy, 2016). Unfortunately, the early period following diagnosis was experienced as the time when provision was at its least regular or provided for assistance with practical (health) solutions to clinical problems. Many people at this point could not ‘accept’ the diagnosis, share their thoughts or socialize with others with Parkinson’s.

**Figure 2:**
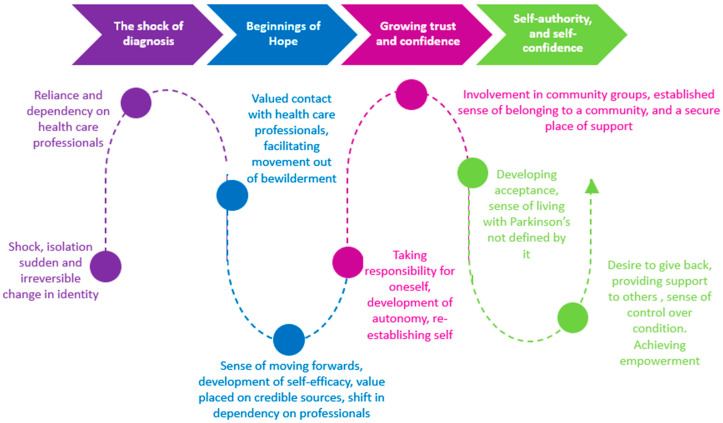
Supporting the journey to empowerment for people with Parkinson’s through the person-centered lens of those living with Parkinson’s [27] (used with permission).

Figure 2 further illustrates factors that PwP considered enabled or hindered successful living with the condition from diagnosis forward, including the role of HCPs [27].

Most PwP wish active involvement to influence how they coped with their condition and to be given agency to be supported through this shared-management model. When they were ready to engage with others, many benefitted from participation in other community and diagnosis-specific groups to help deal with their life-long condition, and from whom they could engage with to gain a ‘working’ understanding of how others lived with the condition [31].

## 7. Towards a New Meaning of Parkinson’s Management

At this moment, there is not a strong enough argument for the need to develop one single approach to self-management but of a recognition from recent research of what self-management should encompass and how it might be delivered. Current models of self-management often prioritize one-off education programmes, which fail to account for the progressive and highly individualized nature of the condition. PwP repeatedly emphasize the importance of self-management support that is phased across the trajectory of the condition, adaptable to changes in physical and cognitive function, and inclusive of their support network [3,31,32].

From the point of diagnosis, PwP often report a sense of disempowerment plus hopelessness leading to demoralization driven by uncertainty, fluctuating symptoms, and difficulties accessing tailored information and support [4,5,27,28]. Early in the condition, individuals are reliant on HCPs for both practical guidance and emotional reassurance. This should be capitalized on by listening to the narrative from PwP for the right education, encouragement, and strategies allowing them to build in confidence, self-efficacy, and a greater sense of agency over time. Crucially, PwP do not want to self-manage in isolation. They request knowledge to be delivered by trained professionals across multiple disciplines who can personalize interventions which additionally integrate essential social and psychological perspectives [4,5,27,28].

PwP value the availability of compassionate HCPs who can provide responsive, expert input when needed—particularly during moments of transition or crisis. Self-management supported by ongoing remote monitoring has been shown to be feasible, acceptable and safe, with the possibility to widen the reach of such programmes not only over longer timespans, but to underserved communities [33].

Rather than perceiving self-management as an endpoint or skill to be acquired, it should be conceptualized as a process—one that evolves in step with the condition and is embedded within a collaborative and ongoing model of care. Shared management approaches, which value the expertise of PwP and their families, alongside professional input, offer a more realistic and empowering pathway. This is particularly important given the limited continuity and duration of professional input available within most healthcare systems [27,28].

Research also highlights that PwP want more than just strategies for managing symptoms—they seek approaches that enable them to reclaim their narrative, maintain roles and identity, and find meaning despite the diagnosis. Initiatives rooted in models such as Positive Health [34] and an awareness of the Disability Paradox [35] provide a valuable framework for supporting such shifts. These approaches recognize that people can experience a good quality of life despite significant symptoms, particularly when supported to focus on their strengths, values, and goals.

Moreover, the diverse presentations of Parkinson’s—whether related to age at onset, gender, phenotype, or psychosocial context—necessitate tailored, multidisciplinary approaches to care. For example, individuals with Young Onset Parkinson’s Disease may require different interventions compared to those with later-onset forms, particularly in relation to employment, parenting, or identity [36]. Importantly, PwP and their families report that when they are ready, peer support groups and community networks can play a vital role in reinforcing a sense of control and shared understanding [31]. They are also more ready than HCPs to encompass newer approaches to ways in which knowledge resources might co-designed and accessed by the community [37].

In redefining Parkinson’s management, it is essential that HCPs move beyond paternalistic models to embrace flexible, person-centered, and relational approaches that acknowledge the evolving capabilities, goals, and contexts of PwP across time.

## 8. Limitations

This opinion piece explores the concept of self-management in Parkinson’s disease, outlining its theoretical underpinnings, key facilitators, and current evidence base. The perspectives presented are informed by existing literature, clinical experience, and close engagement with the Parkinson’s community, which have collectively shaped the issues discussed.

However, it is important to acknowledge that the views expressed are interpretive rather than derived from a formal empirical process. The arguments presented reflect the synthesis of available research and professional insights from healthcare practitioners working closely with people with Parkinson’s (PwP), rather than findings from a systematic or robust research methodology.

We propose that self-management should not be viewed as a singular or static intervention. Instead, it should be understood as a dynamic and responsive process—an agile partnership between PwP and the healthcare professionals best positioned to empower and enable them to live well with their condition. Such an approach must remain flexible, pragmatic, and tailored to the evolving needs and priorities of the individual.

## 9. Conclusions

The concept of self- or shared-management has evolved over the past 3 decades and HCPs need to reconceptualize their role from being the main educator of what Parkinson’s means to the person with the diagnosis into one that empowers the person and their support network to explore what Parkinson’s means to them and how they will best manage their condition over the years.

Findings from the research suggest that effective self-management interventions for PwP requires more than educational content; they demand structured, skills-based training, personalized support, and collaborative partnerships inclusive of social networks.

Empowerment and self-efficacy are key enablers, but must be nurtured through active engagement, contextualized learning, and supportive relationships.

Future research should prioritize rigorous evaluation of delivery models, staff training (e.g., in motivational interviewing), and PwP-identified priorities to better understand what works for whom and why.

## Data Availability

No new data were created or analyzed in this study.

## Abbreviations

CDSMP	Chronic Disease Self-Management Programme
HCPs	Health care professionals
LTCs	Long Term Conditions
PwP	People living with Parkinson’s
QoL	Quality of life
RCT	Randomized Controlled Trial
WHO	World Health Organization
